# GPs’ perspectives on GLP-1RAs for obesity management: a qualitative study in England

**DOI:** 10.3399/BJGP.2025.0065

**Published:** 2025-10-07

**Authors:** Sabrina Keating, Cervantée Wild, Jadine Scragg, Sharon Dixon, Julian Treadwell, Lisa Hinton, Susan Ann Jebb

**Affiliations:** 1 University of Oxford, Nuffield Department of Primary Care Health Sciences, Radcliffe Primary Care Building, Oxford, UK; 2 The University of Auckland Department of Paediatrics Child and Youth Health, Auckland, New Zealand; 3 University of Oxford, Nuffield Department of Primary Care Health Sciences, Radcliffe Primary Care Building, Oxford, UK; 4 NIHR doctoral research fellow and general practitioner, Donnington Medical Partnership, Oxford, UK; 5 NIHR academic clinical lecturer, University of Bristol, Bristol Medical School (PHS), Bristol, UK; 6 University of Oxford, Nuffield Department of Primary Care Health Sciences, Radcliffe Primary Care Building, Oxford, UK

**Keywords:** general practice, glucagon-like peptide 1, glucagon-like peptide-1 receptor agonists, obesity management, qualitative research

## Abstract

**Background:**

Effective treatments are needed for the increasing number of people living with obesity. GPs are key in managing obesity within the NHS but report low confidence in available treatment options. Glucagon-like peptide-1 receptor agonists (GLP-1RAs) have shown promise in weight management, but at the time of this study lacked commissioned primary care service pathways for this indication.

**Aim:**

To explore the perspectives of NHS GPs in England on GLP-1RAs and their integration into primary care for weight management.

**Design and setting:**

In this qualitative study, participants were GPs practising in England, recruited through purposive sampling to reflect diverse geographical and socioeconomic contexts.

**Method:**

Twenty-five semi-structured interviews, which were conducted April–July 2024, were thematically analysed.

**Results:**

Participants generally held positive views about the implementation of GLP-1RAs for weight management in primary care; however, this was joined by hesitations about resource limitations. Navigating consultations with patients asking for prescriptions, or support with private use, often posed difficulties. Concerns included that GLP-1RAs could detract from tackling the broader determinants of obesity. Participants also worried that the medications could be misused, ultimately becoming an overly simplistic solution for patients, practitioners, and the wider health system.

**Conclusion:**

Our findings suggest that while GPs view GLP-1RA integration as a valuable therapeutic option for primary care obesity management, they have concerns about this being done well. To strengthen GP support for implementation, it is essential to recognise the need for adequate resources and ensure that GLP-1RAs are integrated into a holistic strategy for addressing obesity.

## How this fits in

GPs play a central role in managing obesity yet face significant challenges owing to limited treatment options and resource constraints. Glucagon-like peptide-1 receptor agonists (GLP-1RAs) are emerging as a promising treatment for obesity but access in primary care is limited. This study provides new insights into GPs’ perspectives on the integration of GLP-1RAs into primary care, highlighting concerns around resource limitations, health equity, and misuse of the medications.

## Introduction

The proportion of adults living with obesity in England has increased rapidly in the past few decades, from 15% in 1993 to more than one-quarter of the adult population in 2019.^1^ Attempting to lose weight is common, with approximately half of UK adults trying to lose weight at any given time.^2^ Effective treatments are urgently needed to reduce the ill-health associated with obesity.

General practice serves as the entry point to obesity care within the NHS. A range of services now exist including social prescribing, behavioural interventions, and orlistat (a medication that reduces fat absorption).^3^ Evidence suggests that GPs lack confidence in the treatment options they can offer to patients and their effectiveness, resulting in obesity care being perceived as having limited utility.^4–7^

Patients can also be referred to specialist weight management services involving multidisciplinary care team (MDT) management (tier 3), or bariatric surgery (tier 4).^8^ Many localities lack commissioning for these services, and others are unable to meet patient demand, with long-waiting lists that act as a barrier to care.^8,9^

Glucagon-like peptide-1 receptor agonist (GLP-1RA) medications have been described as a ‘game-changer’ within obesity management and at the time of this study were commissioned for use in secondary care.^10–12^ Initially approved in 2005 for the management of diabetes, GLP-1RAs were approved in 2020 to treat obesity in tier 3 services.^13^

GLP-1RAs facilitate weight loss by prompting hormonal changes to increase satiety and slow gastric emptying.^14^ Clinical trials of GLP-1RAs with adjunct behavioural support have demonstrated weight loss of over 15% in people with obesity, although weight may be regained following cessation.^15–18^

In England, at the time of this study, GLP-1RAs could be prescribed to people living with obesity within tier 3 or 4 NHS services, where clinical monitoring and accompanying behavioural support is overseen by an MDT.^14^ However, many patients have reported difficulty in accessing these medications because of limited access to tier 3 services, lack of commissioned prescribing pathways, and national shortages of the medications.^19–21^

This study was conducted before the December 2024 National Institute for Health and Care Excellence (NICE) approval of tirzepatide for obesity management in primary care,^21^ and therefore captures a period during which primary care integration remained uncertain. During the data collection period (June 2024), NICE published a draft guidance consultation indicating the plan for primary care integration.^22^

NICE has since accepted that a phased implementation period will be needed, starting with patients accessing specialist weight management services. From 23 June 2025, integrated care boards have been required to facilitate the prescribing of tirzepatide in primary care for an eligible cohort of patients living with obesity.^23^ Patient eligibility has been prioritised based on highest clinical need, with definition based on comorbidities and body mass index (BMI). It is not yet clear how the implementation will impact primary care staff, patients, and services.^21^

GLP-1RAs have received extensive media and social media attention, prompting individuals to actively seek out these medications through both the NHS and private routes, including online pharmacies, private services, or unregulated providers.^11,19,24^ However, some clinicians have expressed concerns that reliance on medication fails to account for the social, cultural, and environmental factors that inform the pervasiveness of obesity.^24,25^ The aim of this study was to explore the perspectives of NHS GPs in England on GLP-1RAs and their integration into primary care for weight management.

## Method

Participants were deemed eligible if they were currently working as GPs within the NHS in England. The sample was limited to GPs, rather than broader general practice staff involved in obesity care, to address the heterogeneity in roles, prescribing qualification, and staffing within general practices.

The research team included qualitative researchers, GPs, and mixed-methods researchers with expertise in obesity. During the study design process, the research team was aware of growing patient-based demand for GLP-1RAs, as well as concerns regarding the potential workload challenges of integration into primary care.

A patient and public involvement (PPI) panel of three individuals with lived experience of obesity, two of whom had been prescribed GLP-1RAs, contributed to the study throughout. The panel provided insight alongside the wider project team on the topic guide, recruitment strategy, preliminary analysis, and presentation of the data.

Data collection and analysis were led by the lead author, a qualitative researcher with experience in primary care research, but limited prior engagement with research on obesity. The project team and PPI panel provided input on the following: clinical elements of obesity management in general practice; situating study findings within obesity research; and how the findings resonated with lived experience of receiving obesity care. The project team and PPI panel members each held a variety of views regarding GLP-1RAs and obesity management more broadly, allowing for a diversity of perspectives and ways of viewing the data.

Recruitment materials were circulated through multiple channels, including the Society for Academic Primary Care (SAPC) and Royal College of General Practitioners (RCGP) special interest groups, Deep End Networks (practices serving severely deprived populations), and social media. Snowball sampling was also used, with study participants being asked to pass on recruitment materials to other GPs who may be interested in taking part. Further information on recruitment is provided in Supplementary Information S1.

Potential participants self-selected by responding to an email address listed on the recruitment materials. Those who responded via email were asked to fill out a brief online screening form. The form was used to determine eligibility and to consider the demographics of the individual and their practice population. This information was used to inform a purposive sample based on capturing variation across GP practice geography and deprivation levels. Practice postcodes were cross-referenced with scores from the 2019 Index of Multiple Deprivation (IMD). Participants were also asked to report the ethnic and socioeconomic makeup of their practices during the interviews to provide a more in-depth view of the communities they served.

Sample size was determined using information power, which considers the study aims, dialogue quality, and sample specificity, and was iteratively evaluated during data collection.^26^ Recruitment was adapted as needed to include underrepresented geographic regions and deprivation levels. Sampling was finalised at 25 following consensus between the project team and PPI panel that the goals for the sample had been met.

Interview data were collected between April and July 2024. The interviews followed a semi-structured topic guide (Supplementary Information S2) to allow for consistent areas of inquiry while maintaining flexibility in the direction of questioning. The research team’s prior knowledge informed the study topic guide’s focus on gathering ongoing experiences of navigating patient requests for GLP-1RAs. PPI also provided input to the content of the topic guide, yielding additions including question prompts around off-label prescribing of GLP-1RAs and weight management in the context of patients’ broader health. The topic guide was adapted following the publication of the NICE draft guidance on tirzepatide^27^ (published 4 June 2024) to acknowledge the provisional plans for delivery within primary care and to collect participants’ views in light of this update.

Interviews were conducted over Microsoft Teams and lasted from 20–57 minutes. Auto-generated transcripts were checked against the audio-recording to ensure verbatim transcription. Transcripts and study data were encrypted and stored on a secure network drive in line with departmental policy. Summative notes were written up by the lead author after each interview to encourage reflection as interviews were ongoing.

The topic guide and summative notes were used to inform the development of a coding framework, which was created by the lead author with input from the wider project team. The coding framework was applied to each transcript within NVivo (version 12). Analysis utilised the One Sheet of Paper (OSOP) method, wherein data is summarised and grouped within a conceptual mind map with thematic analysis.^28^ The lead author produced the conceptual maps (OSOPs), which were used to derive themes. The themes were then refined through input from the project team and PPI panel to ensure that they clearly and thoroughly captured the interview data.

## Results

Twenty-five GPs currently working in England took part in interviews. A sample overview is provided in Table 1. Participants were based throughout England and worked in areas with varying levels of deprivation. Years in practice ranged from 2–45, with variations in roles including partners (six), salaried GPs (13), and locum GPs (six). Some participants’ views were informed by experience in additional roles, including as a private practitioner, a GP in tier 3 services, an integrated care system diabetes and obesity lead, and a local medical committee medical director.

**Table 1.** Participant demographics

Participant number	Geographical region of England	Practice Index of Multiple Deprivation score^a^	Gender	GP role	Years in practice
INT01	West Midlands	3	Male	Salaried	3
INT02	South East	6	Male	Partner	5
INT03	Greater London	3	Female	Salaried	2
INT04	Greater London	6	Female	Salaried	2
INT05	Greater London	2	Female	Salaried	5
INT06	Greater London	6	Male	Salaried	3
INT07	Greater London	1	Female	Locum	9
INT08	West Midlands	7	Female	Salaried	7
INT09	Yorkshire and Humber	3	Male	Salaried	3
INT10	North West	9	Female	Partner	13
INT11	South East	8	Non-binary	Salaried	6
INT12	Yorkshire and Humber	3	Female	Locum	12
INT13	East Midlands	3	Female	Locum	11
INT14	East	1	Female	Salaried	11
INT15	East	2	Female	Salaried	9
INT16	North East	3	Female	Partner	12
INT17	South West	9	Male	Partner	28
INT18	West Midlands	4	Male	Salaried	45
INT19	North East	1	Female	Partner	23
INT20	East Midlands	4	Male	Salaried	3
INT21	South West	9	Female	Partner	6
INT22	South West	9	Female	Locum	24
INT23	East	10	Male	Locum	5
INT24	South West	N/A^b^	Male	Locum	4
INT25	West Midlands	6	Female	Salaried	3

a The Index of Multiple Deprivation (IMD) ranks local areas in England from most to least deprived. These ranks are divided into deciles, with decile 1 representing the 10% most deprived areas and decile 10 the 10% least deprived.

b Some GPs in the dataset worked across multiple practices in the same region. Participants reported the postcode of the practice they considered their primary affiliation, unless they felt that they did not have one.

Participating GPs reported differing levels of experience in weight management and described varying awareness of the emerging evidence base on GLP-1RAs for weight loss. Generally, participants viewed the safety and efficacy of GLP-1RAs for weight management favourably. Participants often referred to their existing knowledge of managing the medications for diabetes, noting that this familiarity would assist their use for weight management. However, some participants felt more evidence was needed on long-term outcomes and weight regain following medication cessation.

**Figure 1.**
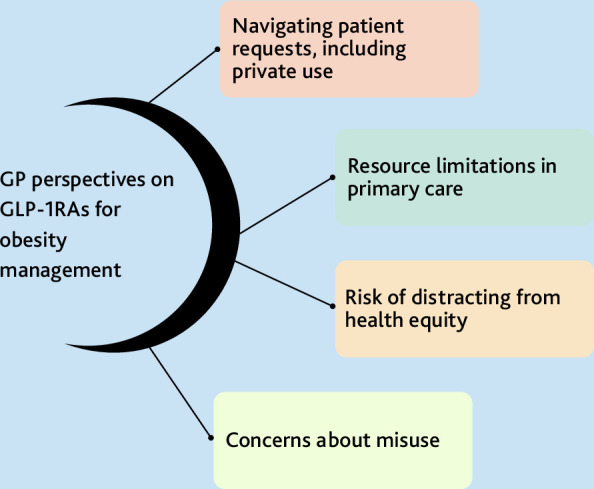
Visual summary of themes. GLP-1RA = glucagon-like peptide-1 receptor agonists

Participant views are presented through the following themes of: navigating patient requests, including private use; resource limitations in primary care; risk of distracting from health equity; and concerns about misuse (Figure 1).

### Navigating patient requests, including private use

Most participants had encountered patients requesting treatment with GLP-1RAs and were aware of patients using them privately. Patients were often unaware that GPs were not commissioned to prescribe GLP-1RAs for weight loss, which produced the uncomfortable dynamic of GPs being the *‘gatekeepers*’ (INT25) of highly desired medications.

*‘Patients often don't understand that I have to work within a formulary ... they think if they try and convince me enough that I will crack and give in, so as soon as they start off on that path, a little part of me dies inside.’* (INT23)

GPs typically responded to GLP-1RA requests by explaining their inability to prescribe and redirecting patients to options such as lifestyle change, orlistat, or (where available) specialist services. Participants expressed discomfort about being unable to meet patient requests and often felt pessimistic about the limited range of alternative options they could offer. Further, participants noted that patients sometimes seemed to have their minds set on receiving medication, with many having already tried orlistat.

*‘If someone is coming in wanting a drug, then it’s hard for me to then say, “right, let’s look at your diet, what you're having,” because that’s not what they came in with and they're not motivated on that side of things.’* (INT01)

A few participants reported responding to requests by having patients assessed for type 2 diabetes, as this could open a different route to accessing GLP-1RAs. It was a source of frustration for some that GLP-1RAs could only be prescribed for patients who already had diabetes, rather than being able to utilise the medications in a preventive role. They felt the options for obesity treatment lacked efficacy, were difficult for patients to access or engage with, or had often already been explored. This resulted in pessimism that patients were left to gain more weight or develop weight-related comorbidities before they could access treatment.

*‘We're doing all this type 2 diabetes work and on the other hand, we're told you can't do non-diabetes obesity work … Should I wait 5–10 years till their knees and hips are broken and they do have diabetes?’* (INT13)

While some patients requesting GLP-1RAs were in principle eligible to receive them through tier 3 or 4 specialist services, this was not routinely available and was typically viewed by participants as unrealistic owing to long-waiting times or a lack of commissioned service pathways. Participants therefore felt that they needed to manage patients’ expectations, and in a few cases discussed private treatment options.

*‘I have to manage expectations and say, “Look, we have to try all these other things first. We could refer you for the Tier 3. But at the moment, I don't know how much point there is if you want this particular intervention because it’s just not available.”’*(INT03)

Most participants reported discomfort around private prescribing, typically from online pharmacies, because of concerns about lacking oversight and the perceived risk of acquiring, or being delegated, liability for medications they had neither sanctioned nor prescribed. This conflict often arose when private prescribers or services contacted the GP— typically after issuing the prescription — asking if there were any known reasons the patient might be unsuitable or ineligible. Participants also described patients requesting advice or monitoring for ongoing use. Supporting private prescribing was viewed by many as a suboptimal use of limited staff time and practice funds, and as potentially unsafe, leading some to refuse to address them.

*‘We've had requests from that* [private prescribing] *company to say “Is this patient safe to use it? Can you look through their records, can they use it?” And as a practice we've said “No.” We're not offering that as a service because it’s extra work for someone that’s not been properly vetted.’* (INT20)

Safety checks by online services, which relied on patient self-reporting, were a common cause for concern, with some recounting cases where GLP-1RAs were prescribed despite contraindications. A few participants worried that patients were accessing the medications through *‘dodgy means’* (INT25) from unregulated sources, potentially putting their safety at risk. Private prescribing also raised concerns about the exacerbation of health inequities, as affluent patients would be more able to pay.

### Resource limitations in primary care

GLP-1RAs were described by many as of benefit to weight management in primary care, as they would add another *‘tool in the toolbox’* (INT18) and could potentially increase the number of patients presenting about obesity. The increase in patient-led presentations was sometimes viewed positively, providing opportunities that may not arise otherwise to discuss weight in a manner agreeable to patients. Some reflected that having GLP-1RAs available could motivate GPs to initiate conversations around weight, as they would feel more able to offer the patient an effective treatment.

*‘One of the reasons that we don't bring* [weight] *up is really in terms of utility … But if it was that somebody was very overweight and they had relevant health issues and we had the option of this medication, I think definitely that would be something.’* (INT23)

While most participants were supportive of the integration of GLP-1RAs into primary care, they were skeptical about whether this could be done well with the resources available. Participants flagged scarce budgets of GP practices, understaffing, long-wait times, and short consultations. A few participants felt that the severity of workforce pressures required prescribing to remain in secondary care.

*‘Every day we are full and oversubscribed, and I just don't see where the capacity to run a primary care therapeutic weight service is, frankly.’* (INT17)

Support alongside GLP-1RA use was viewed as key to creating sustained weight loss but was considered highly resource intensive. Participants worried that relying on medication alone would leave behavioural and psychological factors unaddressed, leaving patients to continue lifestyle patterns that negatively affected their health.

‘[In an ideal world] *there is psychological support alongside, as well as maybe some lifestyle support too … Where the resource for that is, I couldn't say. But I would be wary of* [it just] *being within primary care without* [the] *other support that goes with it.’* (INT19)

Some GPs discussed the integration of GLP-1RAs into primary care in context of the broader *‘creep’* (INT10) of secondary care services into primary care.

*‘A lot of secondary care has moved down into primary care. With the way the weight management services are, people have to wait months and months and months to see someone for something that potentially we could just start in primary care.’* (INT24)

Many worried that the transition into primary care could produce overwhelming patient demand for GLP-1RAs, which would impinge on other patients’ care and the balance of practice budgets. Participants reflected that it was already difficult to prioritise obesity over other health needs, given constraints on time and money.

*‘If we had huge demand for people going on to it, that would detract from other appointments that other people might be looking to get as well.’* (INT09)

Many viewed general practice as the preferred setting for GLP-1RA prescribing and management if there was sufficient financial backing, particularly in context of delays for, and patchy availability of, specialist weight services. Participants highlighted that GPs are best placed to understand how decisions about the use of GLP-1RAs to treat obesity could relate to patients’ mental health, physical health, and life circumstances.

*‘It’s probably best in primary care. We can do that psychological support and we know the patient and we know that there’s domestic violence going on and that they come and they’ve got five kids. So we know about all of that other stuff.’* (INT22)

### Risk of distracting from health equity

Most participants felt that GLP-1RAs should not be relied on as a sole solution to obesity, and worried that their use may distract from addressing health inequity and the broader social and commercial determinants of obesity. GPs identified structural issues, such as financial precarity, disparities in education, limited access to exercise spaces and healthful foods, mental health issues, and cultural norms around eating, as core factors in the development and persistence of obesity that medication could not resolve.

*‘I’m not against the use of GLP-1RAs, but I don't think they should be our “Oh, look, we've got these now. We don't have to address anything else.”’* (INT19)

Several participants voiced concern that medications were only able to address obesity downstream, rather than supporting a preventive, upstream approach. Participants frequently suggested that the strategic and financial investment needed for widespread GLP-1RA use would be better placed into public health and social care measures, as these were viewed as more meaningful and sustainable ways of tackling obesity.

*‘*[It's] *the conditions* [in which] *people are living and working which are dooming them to a certain extent. Anything in terms of weight loss, whether it’s medication or a programme, if it ignores these conditions, it’s only ever going to provide temporary solutions.’* (INT16)

Participants also recognised that patients taking GLP-1RAs would continue to operate within the same personal and societal contexts in which they had struggled to lose weight previously. This was of additional concern for those treating practice populations living in severe deprivation, as these circumstances could not be alleviated by medication.

‘[GLP-1RAs] *do nothing for their housing or financial circumstances. But it may help support people who find themselves in a very tricky situation to help support them lose weight ... it is a good tool in the arsenal, but I worry that it’s not the fix.’* (INT18)

While concern about distracting from health equity was common, some participants felt that GLP-1RAs could be of utility in assisting patients whose circumstances meant they may have less ability to alter their behaviours and lifestyles.

*‘There’s things that a slightly more affluent population can gain help with that* [more deprived patients] *have less access to, so I think they really are in need of an intervention like this.’* (INT04)

Within this view, GLP-1RAs added value as an immediate physiological intervention, rather than relying on public health-level change that would take time, political consensus, and resource investment that may not come to fruition.

### Concerns about misuse

Participants frequently voiced the need for careful consideration in deciding who should use GLP-1RAs and negotiating their duration of use.

Some participants voiced scepticism about pharmaceutical companies, as the push for mass-adoption of GLP-1RAs may reflect profit-driven motives rather than aligning with the needs of patients and the priorities of a publicly funded health system. Prescribing GLP-1RAs universally to those eligible was viewed by many as an expensive and overly simplistic approach. While the potential preventive benefits of GLP-1RAs were recognised, most participants felt that more data on weight regain and long-term health outcomes were needed to make an informed evaluation of the costs and benefits.

*‘It’s the end of GDP* [gross domestic product] *if we focus on drugs as the solution to the obesity epidemic. We are bankrupt as a society for sure. The drug companies would absolutely love us to focus on that ... They're not the solution. We can't afford them to be.’* (INT17)

A few participants found weight-loss medications generally objectionable and considered prescribing them as sanctioning unhealthy choices that negated the responsibility of patients to change their behaviours. Some participants also voiced concern that patients requesting or taking GLP-1RAs may be seeking a *‘quick fix’* (INT10,25) that allowed them to continue their current eating patterns and activity levels.

*‘Just bringing it* [GLP-1RAs] *in so everybody could access it if they've got a certain BMI would completely take the onus off patients to be responsible for their own health.’* (INT21)

Other participants expressed worry that prescribing GLP-1RAs would become the default approach to managing obesity for GPs, as the medications could offer an easier and more immediate approach than doing the more intensive work of supporting lifestyle change.

*'If you're busy and someone says, “can I have this?”, well, generally it’s quite safe and yes, it will be effective. But also you're definitely taking the easier route, and I don't think I blame those people … I would definitely be doing it myself as well because we do it with loads of conditions.’* (INT20)

It was commonly highlighted that refusing to prescribe or continue prescribing GLP-1RAs could be a source of tension in consultations, even where this was in-line with guidance. This tension was typically attributed to a patient’s desire to take or continue to take the medications, which was viewed by some as a source of pressure that risked overriding comprehensive shared decision making. Participants worried about needing to play the role of the *‘policeman’* (INT10) when patients did not meet criteria for continued use.

*‘If people are gaining weight, surely you have to say “Look, this isn't working. We need to forget this.” …You're going to get patients that sort of say “No, you have to prescribe it for me, even though I'm gaining weight,” and it becomes a really difficult dialogue.’* (INT10)

Some participants speculated that this could result in doctors acquiescing to the patient’s desire to continue use, rather than risking harm to the patient or the doctor–patient relationship.

‘[Telling a patient] *“I'm afraid you've hit the two-year point, so I'm stopping a prescription.” Oh, my gosh, that’s never going to happen, is it?’* (INT15)

## Discussion

### Summary

Overall, many participants supported integrating GLP-1RAs into primary care but expressed frustrations and concerns during a period where they had limited ability to prescribe the medications for obesity in primary care. The standard response to patient requests was redirection to available treatments, although participants felt that patients often saw medication as the only viable option. Participants worried about the safety and equity of private prescribing. They emphasised the need for accompanying lifestyle support, viewing it as critical for meaningful change. Concerns were raised that GLP-1RAs could distract from health equity and would fail to address social factors. Participants worried about the potential for the medications to be misused, potentially becoming an overly simplistic option for patients, practitioners, and the health system.

### Strengths and limitations

This is the first qualitative inquiry into English healthcare practitioners’ perspectives on GLP-1RAs and their role in the treatment of obesity before their large-scale introduction into primary care. A particular strength of this study is the incorporation of GPs’ views throughout England, working across different geographical areas, deprivation levels, and demographics. By combining these views, the dataset offers a broad view of GP perspectives on GLP-1RAs at a transitional moment in NHS obesity management.

Data analysis was strengthened through the inclusion of project team and PPI panel members with a range of backgrounds and viewpoints regarding GLP-1RAs and obesity care provision, as this allowed for engagement with a variety of interpretations of the data.

The purposive sampling of GPs based on IMD score mapped imperfectly against participant descriptions of practice populations. A more accurate gauge of this was gained through participant descriptions of their practice demographics, which indicate a diverse sample of deprivation levels.

While this study succeeded in recruiting participants with diverse views, this limited number of interviews cannot capture the breadth of opinion in the GP population. We therefore cannot and do not suggest that these are representative; however, they add valuable perspectives and nuance to a fast-evolving and topical area of care. Further, GPs self-selecting likely steered the sample towards those with interest in the subject.

### Comparison with existing literature

Other studies have explored patient and primary care clinician perspectives on GLP-1RAs for weight loss in the US, a predominantly private healthcare system, and in Denmark, a publicly funded universal healthcare system with out-of-pocket payment for GLP-1RAs at the time of the study.^29,30^ Both describe a clinician-reported increase in patients presenting about their weight, as well as the shift towards patients and clinicians viewing obesity as a more treatable condition within primary care. Similarly, this study, although conducted prior to GLP-1RAs being available for the treatment of obesity in primary care, points to a perceived growth in patient-initiated requests for these medications and GP support for the opportunity this presents for conversations about obesity.

The findings of this study highlight the complex relationship between concerns about cost constraints and the benefits of medication for weight reduction in a publicly funded health system with limited resources. In contrast to the Danish and American samples, some participants voiced hesitation about wide-spread prescribing and the provision of GLP-1RAs within NHS primary care in England.

Andreassen *et al*’s study^30^ reported potential issues around shared decision making, with high patient motivation resulting in GLP-1RAs typically being prescribed despite clinician concerns about suitability. Within the present study, concerns regarding shared decision making were also raised around initiation and deprescription.

The present study brings to the fore considerations of where responsibility for addressing obesity should be placed. This is similarly identified in Andreassen *et al*’s work^30^, where participants described an objection to GLP-1RA use for aesthetic reasons, as this negated individual responsibility for their health. Within this dataset, GLP-1RAs were seen as at risk of being framed as an easy way out for a variety of players, including clinicians uncritically prescribing the medications, patients not wanting to change their behaviours, and the NHS in England embracing a short-term stopgap rather than tackling the social and commercial determinants of obesity.

Research repeatedly points to the barriers facing weight management in general practice in England, including a lack of time, resources, training, comfort discussing the subject, and confidence in the treatment options available.^4–7,31,32^ The interviews in this study demonstrated the persistence of these barriers, and suggest some of the ways that GLP-1RAs could both improve and perpetuate them. The lack of time and funding were described as challenges to integrating GLP-1RAs effectively, particularly as demand could negatively affect the care of other patients. This study’s findings also highlight the potential for GLP-1RAs to enable more conversations about weight, as they offer a more immediate treatment.

### Implications for research and practice

Much remains unknown about how to best integrate GLP-1RAs into primary care in England. The rollout of tirzepatide in primary care in the UK has now commenced with a prolonged implementation phase, starting with patients who have a BMI of ≥40 kg/m² and four or more qualifying comorbidities.^22,23^ It is important that there is careful evaluation of this service to optimise next steps.

The findings of this study highlight the need for individual, local, and national approaches to GLP-1RA prescribing to actively prioritise health equity. Additionally, the findings lend emphasis to the need for adequate resourcing to ensure that primary care can meet demand without compromising on monitoring and behavioural support.

Additional knowledge is needed to support the service design, implementation, and economics of GLP-1RA prescription, with attention towards how these will interact with inequity and deprivation. Integration of GLP-1RA prescribing into primary care services will require access to and referral into services offering behaviour change and lifestyle support. GPs are likely to be the first port of call for queries about access to these services and also about medication access and side effects, in a context of already over-stretched services. Further consideration should be given to the added labour of managing requests, hopes, and expectations from patients both eligible and ineligible for treatment, particularly in light of the phased roll-out and media attention surrounding these medications.

Additionally, research is required to understand patient experiences of the medications and what support they need from primary care. GLP-1RAs may present specific challenges regarding initiation and deprescribing, creating a need for work on navigating shared decision making.^30^ Further research on clinician perspectives on GLP-1RAs is also indicated, given the rapidly evolving context of use and knowledge.
